# Supplementary material for: Orthostatic stability with intravenous levodopa
Source: PeerJ. 2015 Aug 27;3:e1198. doi: 10.7717/peerj.1198 (PMC4556150; doi:10.7717/peerj.1198)
Supplement: Supplemental Information 2 [file peerj-03-1198-s002.docx]

| 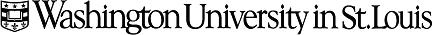 | Form 2 Biomedical 8/08 Human Research Protection Office [x] Adult [] Minor |
| --- | --- |

**INFORMED CONSENT TO PARTICIPATE IN A RESEARCH STUDY**

| Participant’s Name |  | HRPO # | **05-0832** |
| --- | --- | --- | --- |

| Principal Investigator | **Black, Kevin J. M.D.** | PI’s Phone Number | **(314) 362-6514**  **(314) 362-7651** |
| --- | --- | --- | --- |

^Last First Credentials^

| Title of Project: | **Dopaminergic effects on cortical function in Tourette’s**  **(levodopa study)** |
| --- | --- |

You may be eligible to take part in a research study. This form gives you important information about the study. It describes the purpose of the study, and the risks and possible benefits of participating in the study.

Please take the time to review this information carefully. After you have finished, you should talk to the researchers about the study and ask them any questions you have. You may also wish to talk to others (for example, your friends, family, or other doctors) about your participation in this study. If you decided to take part in the study, you will be asked to sign this form. Before you sign this form, be sure you understand what the study is about, including the risks and possible benefits to you.

1. **Why is this study being done?**

The overall purpose of this research is to determine whether the brain performs memory and decision-making tasks differently in Tourette Syndrome (TS). TS is a movement disorder characterized by vocal tics (sounds) and motor tics (movements). We will measure how and where brain activity changes using magnetic resonance imaging (MRI) scans during memory tasks and after taking levodopa. Levodopa is a drug commonly used for the treatment of Parkinson’s disease (PD).

Being in a research study does not take the place of routine physical exams or visits to your own doctor and should not be relied on to diagnose or treat medical problems.

1. What am I being asked to do?

During the screening, you will have an evaluation that will include questions about your physical and mental health and a neurological and medical examination. We will also ask questions about your school and work experience. If you have tics, we will ask you several questions about your tics as well as other symptoms sometimes associated with TS. If you have TS, you will also be asked to fill out questionnaires regarding your tics and other symptoms. If you do not have TS, you will not be asked to fill out these questionnaires since they would not apply to you. The questionnaires could take from 30 minutes to an hour to complete. Also, you will be asked to complete some memory and thinking tests.

You may be asked not to take certain medications on the day of the study. The investigator or coordinator will discuss your medications with you. For some medications (for instance Prozac), you may need to stop the medicine for more than a day. We will review your current medications with you and give you instructions for your specific situation. If you are a woman of childbearing age, you may be asked to take a pregnancy test before each study day. If you are pregnant, you may not participate.

On each study day, you will be asked to not eat any breakfast or drink any caffeine. This is meant to help prevent an upset stomach by the levodopa and to prevent the food’s interfering with the experiment. When you arrive, you will be given carbidopa tablets (a medication commonly used in treatment of PD) which will help to prevent an upset stomach caused by the levodopa. A small plastic tubing (catheter) will be placed in a vein in one arm using a small needle to establish an “IV” (intravenous) line.

Levodopa is a medicine commonly used to treat Parkinson’s disease. Levodopa in pill form is approved by the United States Food and Drug Administration (US FDA). However, giving levodopa through a vein is not FDA approved, though over 1500 people have received IV levodopa over the past 40 years.  Since this method of giving levodopa is considered investigational, we have requested special permission from the FDA to give levodopa through an IV.  Additionally, we have given IV levodopa over 150 times at our facility with no serious adverse reactions.  There are no known additional side effects of levodopa when given through an IV rather than by mouth, though there could be risks that are unknown.

We will infuse levodopa or saline (an inactive substance sometimes called a placebo) through the IV. During one visit you will be given levodopa and on the other, saline. On the study day, neither you nor we will know whether you got levodopa or the inactive substance (placebo). We will find out later. This is important because if you knew which one you were getting, your brain might respond differently just because of expectations you might have about possible effects of levodopa.

After the infusion starts, you will then have MRI (Magnetic Resonance Imaging) scans. This scan uses strong magnets and radio waves to produce scans that show the structures in the brain and the activity of the brain. The MRI scanner is a large tube that covers your body. You will be required to lie completely still within the MRI scanner for several minutes at a time. Your total time in the scanner at each visit could be up to 3 hours. Halfway through the study we will ask if you want to get out of the scanner for a break.

You will be asked to perform several memory tasks while we are scanning your brain. The memory tasks require you to remember letters or locations of objects that appear on a screen. You will press buttons when you see certain letters or objects.

While you are lying in the scanner, you will be videotaped so that we can see possible movements or tics while you are lying in the scanner. The videotapes will be labeled only by a number and the date of your study, to help protect your identity. However, the videotapes will be identifiable, meaning that your tape can be linked to your name. This is necessary for the research team to be able to compare the videotapes to the scan data from the MRI. The videotapes will be kept in a locked office in a locked suite and only available to members of the research team. We may keep these videotapes for up to 10 years after the study ends.

Blood samples will be taken from the other arm using a small needle before and after the MRI. Less than 2 tablespoons of blood will be taken total. These samples are taken to determine the levels of levodopa and carbidopa in your system.

After the MRI is completed, you will complete a computerized decision-making task and we will ask several questions about how you are feeling. If you have tics, we will also ask questions about your symptoms. We will remove your IVs and make sure that you are feeling okay. Then, you will be given lunch and be free to leave. After the study is over, you are free to take any medication that you had been asked to skip for the morning of the study.

All procedures involved in this study are undertaken solely for the purpose of research.

The following table summarizes your participation:

Visit Purpose Time required

Screening to determine whether or not you can participate up to 2 hours

Day 1 to test memory after levodopa (or saline) up to 4 hours

while having your brain scanned;

to test decision-making after levodopa (or saline)

Day 2 to test memory after saline (or levodopa) up to 4 hours

while having your brain scanned;

to test decision-making after saline (or levodopa)

Day 3 if recommended up to 4 hours

**How long will I be in the study?**

The study includes a screening, which will take 1-2 hours, and 2 or 3 MRI days for up to 4 hours per study day if you qualify. Most participants complete the study in two MRI days. (This works out to a maximum of 14 hours of participation.)

1. **What are the costs?**

There will be no charge to you or to your insurance company for participating in this research.

**Will I be reimbursed for my participation?**

Volunteers will be compensated $25 per hour for participation in this study for their time and inconvenience. If you complete the entire study, this will amount to about $200-$350 depending on how long the screening and study days take. If you do not complete the entire study, you will still be paid on an hourly basis.

1. **What are the risks?**

**Likely:**

The placement of the IV needle into the vein may be uncomfortable. There is a potential of blood clot (bruise) forming under the skin.

The memory and decision-making tasks may be slightly boring, fatiguing or challenging.

Some people get muscle aches and pains from lying on their back. This will be minimized by providing cushions at pressure points and beneath the knees as desired. Earplugs or headphones will be used to dampen the sound of the MRI procedure.

Because you are asked to skip breakfast, you may notice hunger the morning of the study.

For TS subjects who go without tic or stimulant medication the morning of the study: transient worsening of ADHD symptoms.

**Less likely:**

Levodopa and/or carbidopa have a small chance of causing temporary nausea, vomiting, or lightheadedness. Should this occur, medication is available to minimize these symptoms.

Some people may feel lightheaded or faint when the IV catheter is placed. There is a slight chance of infection.

If you were asked to miss a dose or doses of an antidepressant medication such as Prozac or Paxil, you could notice temporary dizziness.

Some people feel claustrophobic in the scanner. If you feel claustrophobic and we cannot help you feel comfortable, we will end the experiment. We will also end the experiment at any time if you ask us to.

**Rare:**

The questions that you are asked during this study could make you feel uncomfortable. If any question makes you feel uncomfortable, you may choose not to answer it.

Very rarely, levodopa can cause involuntary movements in normal subjects. Rarely, hallucinations (seeing or hearing things that are not really there), delusions (having strange thoughts that others would disagree with), or mental confusion could occur. If any of these do occur, they are temporary. There is a rare possibility of an allergic reaction to levodopa. Also, because I.V. administration of levodopa is considered experimental, there may be unexpected side effects. While none have been encountered to date at Washington University, and we know of none occurring elsewhere, these could be serious or even fatal. You will be told of any new information that may affect your willingness to participate in this study.

Very rarely, levodopa can cause temporary decrease in blood pressure.

If you have some kinds of metal in your body (such as a pacemaker or aneurysm clip) and failed to disclose that information to the study personnel, then an MRI scan could be extremely dangerous to you.

One potential risk of participating in this study is that confidential information about you may be accidentally disclosed. We will use our best efforts to keep the information about you secure, and we think the risk of accidental disclosure is very small. Please see the Confidentiality section of this consent form for more information.

You may experience all or some of the risks listed above. There may also be unknown risks. The PI will answer any questions you have about these risks.

- **Certificate of Confidentiality**

One potential risk of participating in this study is that confidential information about you may be accidentally disclosed. We will use our best efforts to keep the information about you secure, and we think the risk of accidental disclosure is very small. Please see the *Confidentiality* (Item 7) section of this consent form for more information.

- **Coded Data**

The information you give us will be given a code number. A master list linking the code number and your identity will be kept separate from the research data. Only the PI and people helping him/her will be able to see the list. We will protect your information, but there is a chance somebody might see it.

#### Pregnancy/Childbearing Potential

**If you are a woman of childbearing potential, please read and sign below.**

Some research medications or procedures can cause severe birth defects and mental retardation to a fetus (unborn baby). If you are pregnant, think you are pregnant, or if at any time during your study participation there is a lapse in your birth control procedures, it is important for you to notify the investigator immediately.

If you participate in a study that includes a drug or procedure, you must be willing to: have a pregnancy test done at the discretion of the investigator before beginning your participation; avoid becoming pregnant during your participation; and agree to use a method of birth control that is acceptable with the investigator.

Your signature below indicates your agreement to comply fully with these requirements.

 Signature Date

It is important that you inform the study doctor if you are a lactating female (actively breast feeding).

#### Sexually Active Male

**If you are a sexually active male, please read and sign below.**

Some parts of this study might cause an unborn baby to have physical or mental problems.

You and your partner must agree to use birth control if you want to take part in this study. Notify the PI if your partner becomes pregnant or if your birth control method fails while you are on the study.

Please discuss with your research physician whether you need to wait for a certain period of time after the study is over before fathering a child.

By signing below, you agree to follow these rules**.**

Signature Date

What happens if you are injured because you took part in this study?

Washington University investigators and staff will try to reduce, control, and treat any complications from this research. If you feel you are injured because of the study, please contact the investigator, Kevin Black, M.D. at (314) 362-6514 and/or the Human Research Protection Office Executive Chairperson, Dr. Philip Ludbrook at (314) 633-7400 or 1-(800)-438-0445.

Decisions about payment for medical treatment for injuries relating to your participation in research will be made by Washington University. If you need to seek medical care for a research-related injury, please notify the investigator as soon as possible.

1. **Are there benefits to taking part in the study?**

If you are a subject with TS, you may experience brief improvement in your tics if you receive the active drug. There are no other direct benefits to you for participation in this research. The possible benefits to society from this research are: that this study is intended to increase our understanding of how the brain functions in people with and without TS. In the future, these studies may aid physiological or genetic studies, or clinical management of TS, or other illnesses in which dopamine function is abnormal.

1. **What other options are there?**

There are no alternatives, other than not participating in the research. Taking part in this research study is voluntary. You may choose not to take part in this research study or you may withdraw your consent at any time. You may withdraw by telling the study team you are no longer interesting in participating in the study or you may send in a withdrawal letter. A sample withdrawal letter can be found at <http://hrpo.wustl.edu> under Information for Research Participants. Your choice will not at any time affect the commitment of your health care providers to administer care. There will be no penalty or loss of benefits to which you are otherwise entitled.

1. **What about privacy and confidentiality?**

We will do everything we can to protect your privacy. Protected Health Information (PHI) is health information that identifies you. PHI is protected by federal law under HIPAA (the Health Insurance Portability and Accountability Act). To take part in this research, you must give the research team permission to use and disclose (share) your PHI for the study explained in this consent form.

A Certificate of Confidentiality (COC) has been obtained from the Department of Health and Human Services. This will help further protect information that may identify you. The Certificate prevents the investigator from being forced to disclose identifying information for use in court. The investigator may not even be forced by court subpoena. Courts that may be prevented from getting your information include any federal, state, local, civil, criminal, administrative, legislative, or other court proceeding.

You should understand that the COC does not prevent you or a member of your family from voluntarily releasing information about yourself or your involvement in research. The investigator may not withhold information if you give your insurer or employer permission to receive information about your participation in this research. This means that you and your family must also actively protect your own privacy.

The Certificate does not prevent the researchers from taking steps, including reporting to authorities, to prevent serious harm to yourself or others. Such disclosures will be made as described below.

In addition to health information that may be created by this study, the research team may look at your clinic/physician records if you are a patient of the Movement Disorders Center at Washington University School of Medicine. This record may include pathology or radiology results or lab tests, e.g. blood tests, in addition to clinical notes. We will record information needed for the study in your research file.

Your research record is separate from your medical record and access to your research data will be handled on a case by case basis. If any abnormalities were found in your brain scans, we would tell you and, with your permission, alert your regular doctor. We would also make the relevant brain scans available to your doctor on your request.

**The** **research team may share your information with:**

- The Department of Health and Human Services (DHHS) to complete federal responsibilities for audit or evaluation of this study.
- Public health agencies to complete public health reporting requirements
- Hospital or University representatives, to complete Hospital or University responsibilities for oversight of this study.
- Your primary care physician if a medical condition that needs urgent attention is discovered
- Appropriate authorities to the extent necessary to prevent serious harm to yourself or others.
- The Food and Drug Administration (FDA)

Once your health information is shared with someone outside of the research team, it may no longer be protected by HIPAA.

The research team will only use and share your information as talked about in this form. When possible, the research team will make sure information cannot be linked to you (de-identified). Once information is de-identified, it may be used and shared for other purposes not discussed in this consent form. If you have questions or concerns about your privacy and the use of your PHI, please contact the University’s Privacy Officer, at 866-747-4975.

We will be keeping data from this study for up to 10 years after the study ends. We may look back at this data for future studies of fMRI imaging or TS. All information will be kept in a locked office in a locked suite and only available to members of the research team. We will code your data by a research number only so that there is no identifying information to link you to the data.

May we keep your data (other than video) for future research studies of levodopa, TS or brain imaging?

Please initial your response Yes_______ No________

May we share your research data (with no identification except code numbers) with

investigators doing research in similar fields at Washington University and at other research centers? Other investigators will NOT receive your name or any other identifying information.

Please initial your response. Yes ________ No ________

| This study is sponsored by The National Institute of Health. Representatives of the sponsor will have access to your research and/or medical records for monitoring the study. The research team will also send study results to the sponsor. Information sent to the sponsor will be deidentified. The sponsor is not required to abide by the HIPAA regulations, but agrees to protect the confidentiality of your information. |
| --- |

**If you decide not to sign this form, it will not affect**

- your treatment or the care given by your health provider.
- your insurance payment or enrollment in any health plans.
- any benefits to which you are entitled.

However, it will not be possible for you to take part in the study.

**If you sign this form:**

- You authorize the use of your PHI for this research
- Your signature and this form will not expire as long as you wish to participate.
- You may later change your mind and not let the research team use or share your information (you may revoke your authorization).
  - To revoke your authorization, complete the withdrawal letter, found in the Participant section of the Human Research Protection Office website at [http://hrpo.wustl.edu](https://hrpo.wustl.edu/HRPO/Rooms/DisplayPages/LayoutInitial?Container=com.webridge.entity.Entity%5bOID%5bAD20143D90854C428C91E4295D5C72FE%5d%5d) (or use the direct link: <http://hrpohome.wustl.edu/participants/WithdrawalTemplate.rtf>) or you may request that the Investigator send you a copy of the letter.
    - **If you revoke your authorization:**
      - The research team may only use and share information already collected for the study.
      - Your information may still be used and shared if necessary for safety reasons.
      - You will not be allowed to continue to participate in the study.

Do you already have contact restrictions in place with WUMC? Yes _____ No_____

(Example – no calls at home, no messages left for you, no –emails, etc.)

Please specify any contact restrictions you want to request for this study only.

________________________________________________________________________________

________________________________________________________________________________

Request to transmit Protected Health Information via e-mail

The research team requests permission to send you questionnaires, appointment confirmations and directions via the e-mail address you provide us.

Samantha Blankenship and Mary Creech in our office will have access to your e-mail communications. You may e-mail our office at _blankenships@npg.wustl.edu_. This e-mail address is checked (list how often) but should not be used for emergency or urgent contacts. We will only be using this e-mail address to send you the information listed above. If you have any questions or need to contact us for an urgent or emergent situation, please contact our office at _(314) 362-6514 or (314) 362-7651_.

E-mail correspondence is kept as long as the research record is kept, 10 years.

You should be aware that there are risks associated with sending protected health information via e-mail and the internet. Risks include:

Use of unsecured computers – When using any public computer you should be careful to protect your username and password. Make sure you log-out before getting up from the computer. Do not use any public computer where you feel uncomfortable.

Use of home computers – If you share a home computer with other family members, make sure you identify an e-mail address that only you can access. Make sure that you log out of the computer before getting up and letting another family member use it.

Use of work computers – Your employer will have access to any e-mail communications sent or received on a work computer.

Internet risks – Information being sent to you from our office is being sent via a secure server. This means that the information will be encrypted. However, there is always a risk that the message could be intercepted and decoded or sent to the wrong e-mail address. To avoid sending messages to the wrong e-mail address, the first e-mail we send you will be a test message to ensure we have the correct e-mail address.

Do you agree to allow us to send you protected health information via e-mail? ___ Yes ___ No

**Notice of Privacy Practices**

| This consent form or similar documentation that you are participating in a research study will be included in your clinical medical record. Anyone with access to your medical record, including your health insurance company will be able to see that you are participating in a research study.  The Notice of Privacy Practices is a separate document. It describes the procedures used by WU to protect your information. If you have not already received the Notice of Privacy Practices, the research team will make one available to you.  ______ I have been offered a copy of the Notice of Privacy Practices.  ^Initial^ |
| --- |

1. **Whom do I call if I have questions or problems?**

## Please contact the researcher listed below to:

- Obtain more information about the study
- Ask a question about the study procedures or treatments
- Report an illness, injury, or other problem (you may also need to tell your regular doctors)
- Leave the study before it is finished
- Express a concern about the study

Principal Investigator:_Kevin J. Black, M.D.__

Mailing Address:_Box 8225 660 South Euclid Ave. St. Louis, MO 63110_

Telephone:_(314)362-6514_

If you wish to talk to someone else, or have questions or concerns about your rights as a research participant, call Dr. Philip Ludbrook, Executive Chair of Washington University’s Human Research Protection Office (WU HRPO) at (314) 633-7400, or 1-(800)-438-0445.

1. The Principal Investigator (PI) may withdraw you from the study without your consent if

considered appropriate. It may be in your best interest to allow follow-up outside the study. For instance, if you experienced severe nausea or did not keep appointments, the PI might decide to discontinue your participation. The PI will share any new information that could change how you feel about continuing in the study.

1. You will be given a signed copy of this consent form for your records.

**Research Participant:**

I have read this consent form and have been given the chance to ask questions. I agree to participate in this research described above, titled: Dopaminergic effects on cortical function in Tourette’s.

HRPO does not require participants to re-sign the consent form unless a change is made; the investigator, however, may choose to re-consent participants at any time.

Signature:

Printed Name: Date of Signature:

**Principal Investigator (or Designee):**

I have given this research participant (or his/her legally authorized representative, if applicable) information about this study that I believe is accurate and complete. The participant has indicated that he or she understands the nature of the study and the risks and benefits of participating.

Signature: Title:

Printed Name: Date of Signature:

**This form is valid only with the Human Research Protection Office’s current stamp of approval.**
